# Minimally invasive total hip arthroplasty with the anterior approach

**DOI:** 10.4103/0019-5413.41853

**Published:** 2008

**Authors:** B Sonny Bal, Santaram Vallurupalli

**Affiliations:** Department of Orthopedic Surgery, University of Missouri, Columbia, USA

**Keywords:** Total hip arthroplasty, minimally invasive surgery, Anterior approach of hip

## Abstract

**Background:**

Total hip athroplasty with the anterior surgical approach is advised because the dissection is entirely within intermuscular planes. In this report we describe a minimally invasive technique of anterior total hip arthroplasty, with the early outcomes.

**Materials and Methods:**

The technique of minimally invasive total hip arthroplasty with anterior approach (Smith-Petersen) is described. We reviewed data on 100 consecutive patients who underwent anterior total hip arthroplasty with uncemented components. Mean patient age was 61 years (range 33-91). Mean patience BMI 29.8 (range 18.1-51.8).

**Results:**

Minumum follow up duration is 10 months. The mean duration of surgery was 53 min (range 34-87) with mean blood loss 185 cc (range 65-630), and the mean incision length was 10.4 cm. Clinical and radiographic outcomes were similar to historical outcomes of standard total hip arthroplasty.

**Conclusions:**

With proper surgeon training, minimally invasive total hip replacement with the anterior surgical interval is safe and efficacious.

## INTRODUCTION

Less invasive surgery, specially total hip arthroplasty (THA) is of interest to surgeons and patients, with the goal of improving early recovery parameters.^1^,^2^ Patients are intuitively attracted to the concept of less invasive surgery, associating it with less trauma and a better cosmetic result.^3^ In skilled hands, total hip replacement performed with two small incisions can lead to earlier hospital discharge and quicker recovery when compared to standard THA using longer incisions.^4^,^5^ Variations of this technique that involves acetabular cup implantation through a modified Smith-Peterson surgical interval, and femoral stem insertion through a separate accessory incision made further posteriorly have shown safe outcomes in large clinical series.^6^,^7^

Despite the technical challenges inherent in performing THA through limited incisions, some surgeons have reported safe and favorable outcomes.^8^,^9^ In this paper, the technique of THA using a single, short modification of the Smith-Petersen surgical interval^10^ is described. Key technical steps, potential pitfalls, and early outcomes in a consecutive series of patients are presented.

## MATERIALS AND METHODS

### Equipment

The method described here requires a special orthopedic table (PRO*fx* or HANA model, OSI, Union City, CA) for patient positioning in the supine position. Minimally invasive total hip arthroplasty (MIS-THA) is facilitated with a motorized lift built into the table. The lift is designed to elevate and expose the proximal femur for stem insertion. Other authors have performed anterior THA without the table, using specialized instruments to facilitate exposure.^11^ The advantage of using a table is that it assists retraction, and enables optimal leg positioning. Lighted Hohman retractors and custom instruments are also useful during MIS-THA through the anterior approach although the procedure can be done with standard instruments as well.

Intraoperative radiography and fluoroscopy can be helpful to guide component positioning during MIS-THA performed with two incisions^12^ and during MIS-THA performed with a single anterior incision. In the technique described below, no X-rays are needed. If the surgeon chooses to use intraoperative fluoroscopy, careful attention should be paid to variations in patient positioning, spinal curvature, and skeletal anatomy of the pelvis.^13^ The alternative method of direct visualization of skeletal anatomy, preoperative radiograph templating, and the use of alignment guides for component positioning can obviate the need for intraoperative fluoroscopy during anterior MIS-THA.

### Operative procedure

#### Patient positioning and draping

**Figure 1:**
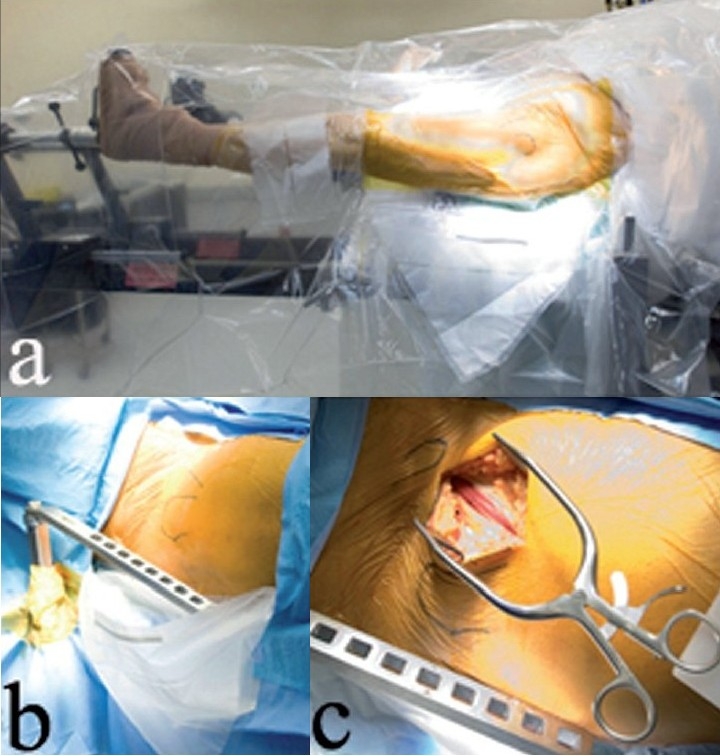
(a) Leg positioning on the HANA orthopedic table is shown, with a fracture drape covering the operative left leg. (b) A right hip is shown draped, with the abdominal drape over the plastic fracture drape, and the hook spar exiting the drapes through a hole. (c) A right hip incision is shown, with teh fascia over the tensor split

Following induction of a spinal anesthesia, the patient is positioned supine on the HANA table. The operative foot is secured properly in the leg attachment to avoid slipping during surgery [Figure 1a]. The ipsilateral arm is folded over the chest. In obese patients, abdominal fat folded over the iliac crest should be retracted using adhesive tapes. The groin crease should be visible during this procedure.

Skin preparation is from the distal third of the thigh to one hand-breadth above the iliac crest [Figure 1b]. Draping can be simplified by using drapes made for hip fracture fixation. First, a betadine-impregnated plastic hip fracture drape is placed on the anterior-lateral thigh. This is followed by an abdominal drape placed over the first drape. The plastic pouch built into the first drape is brought out through the opening in the second drape to capture blood and irrigation fluid during surgery [Figure 1b]. Additional drapes can be used if needed at the margins of the sterile field.

#### Surgical exposure

The anterior superior iliac spine (ASIS) and greater trochanter are identified by palpation. Rarely, if this is not possible, or if the surgeon is uncertain, these anatomic landmarks can be identified with intraoperative X-rays. A straight incision is made obliquely on the anterior-lateral thigh, beginning 2 cm distal and lateral to the ASIS and ending 2 cm anterior to the greater trochanter. Crossing the groin crease with the incision is preferable to forcible skin stretching during stem insertion, which can lead to skin damage and poor healing. Early in the learning curve, it is safer to make a longer skin incision, especially since incision length does not appear to correlate with recovery. Instead, recovery following anterior total hip surgery may be related to the intermuscular dissection, lack of gluteus maximus splitting, and the ability to mobilize the patient without posterior hip precautions. With experience, it is possible to perform the procedure consistently with an 8-12 cm incision length.

**Figure 2:**
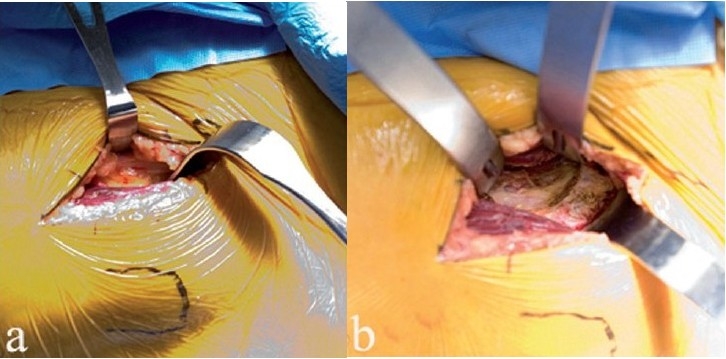
In the left hip shown (a), the tensor is retracted laterally, and the rectus femoris muscle is retracted medially. A cobra retracts the tensor laterally (b), and a Hohman retractor retracts the psoas/rectus femoris medially, exposing the hip capsule. The second sharp Hohman is on the anterior acetabular wall

The subcutaneous fat is dissected bluntly until the thin fascia over the tensor fascia lata muscle is seen [Figure 1c]. Blunt dissection will minimize the risk of injury to the lateral femoral cutaneous nerve which is always at risk during anterior approaches to the hip joint. Any branches of the lateral femoral cutaneous nerve that are visualized in the subcutaneous fat should be retracted laterally. The incision itself should be placed as far laterally on the thigh as possible to minimize the risk to the lateral femoral cutaneous nerve. Next, the fascia overlying the tensor muscle is incised, and the tensor muscle is separated from the sartorius. The tensor muscle is retracted laterally by placing a cobra retractor between the muscle and the superior hip capsule [Figure 2a]. Capsular insertions of the rectus femoris and psoas muscles are elevated and retracted medially with another cobra retractor, or a lighted Hohman retractor [Figure 2b]. A sharp-tipped Hohman retractor is next placed on the anterior acetabular wall to gain exposure. With this exposure, the lateral femoral circumflex vessels can be identified distally, crossing the surgical field. These vessels must be coagulated or ligated to avoid bleeding.

The interval between the tensor and the rectus femoris should be identified and developed distally; this step is especially necessary in heavier individuals. In very muscular patients, partial release of attachments between the tensor and rectus femoris insertions near the ASIS will facilitate exposure. The goal is to avoid injury to the tensor muscle during lateral retraction; one pitfall with this method is inadvertent laceration of the tensor muscle belly from excessive retraction. The surgeon should proceed further only if the tensor muscle can be retracted laterally with minimal tension.

**Figure 3:**
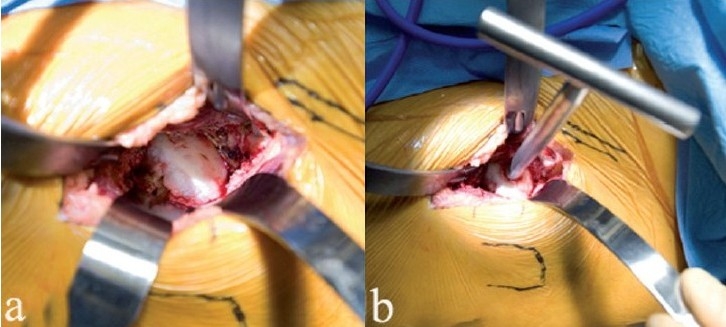
Femoral head exposure (a) after anterior capsulotomy. A corkscrew (b) is placed in the head after maximum external rotation, to facilitate anterior dislocation of the femoral head

The anterior hip capsule is opened with two flaps that are retracted by repositioning the cobra retractors previously placed outside the hip capsule. The femoral head and anterior acetabular wall will come into view [Figure 3a]. A few millimeters of the anterior acetabular wall and calcified labrum/osteophyte complex should be excised with an osteotome since this step will ease insertion of a hip skid and facilitate anterior dislocation of the femoral head. This step is advocated since many hips with osteoarthritis have acetabular retroversion and impingement of the femoral neck, or may have developed osteophytes on the anterior rim in response to degenerative disease. Removal of a few millimeters of the anterior acetabular wall is a technical step that facilitates the procedure.

#### Neck osteotomy and femoral head extraction

**Figure 4:**
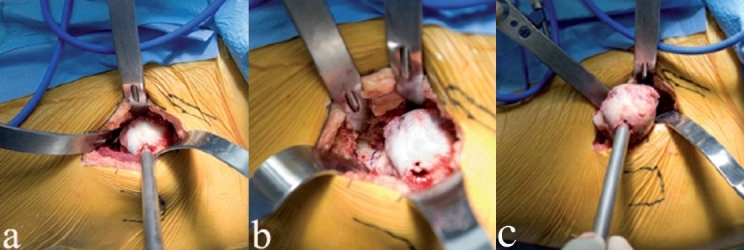
The femoral head is dislocated anteriorly (a) by using a skid in the hip joint, anterior traction on the corkscrew handle, and gentle external rotation of the leg. Removing a few millimeters of the anterior acetabular wall will facilitate this step. With the head anteriorly dislocated, the lesser trochanter and calcar are exposed by subperiosteal elevation of the medial hip capsule (b). The proposed calcar cut has been marked with a pen, in relationship to the lesser trochanter. The corkscrew has been removed to make the calcar cut. The femoral head is removed (c) by cutting the calcar first and then making the lateral cut, thereby avoiding inadvertent injury to the greater trochanter. Another option is to remove a segment of the femoral neck *in situ*, followed by extraction of the head

With slight traction on the leg, a hip skid is placed in the hip joint, and slight external rotation will expose the femoral head. A drill hole is made in the head, with the traction off, and a T-handled corkscrew is driven into the femoral head [Figure 3b]. Leverage from the hip skid, and anterior traction on the T-handle will dislocate the femoral head anteriorly without excessive torque on the leg [Figure 4a].

Difficult anterior dislocation of the hip can lead to forcible rotation of the leg. A pitfall of this method relates to iatrogenic fracture of the femur or ankle from excessive twisting. The safe technique involves proper placement of the corkscrew, anterior leverage with the T-handle, and removal of part of the anterior acetabular wall. If these steps are taken, the femoral head will slip out without forced external rotation of the leg. While iatrogenic fracture of the femur and ankle is a serious risk of the procedure described here, these complications are entirely avoidable and have not been encountered in the authors' experience. If proper steps are followed in sequence, excess torque on the leg should not be necessary in any case, thereby obviating the risk of iatrogenic fracture.

Femoral head dislocation is not necessary. An alternative is osteotomy of the femoral neck at two places *in situ*, followed by removal of the neck segment with a Steinmann pin, and subsequent extraction of the femoral head.^12^ The reason anterior hip dislocation is useful is that it facilitates proximal femur exposure, visualization of the lesser trochanter, and release of tight posterior tissues. Only one assistant is needed during surgery; this person stands on the side opposite the operated hip. In difficult cases, or if available, a second assistant can be positioned on the ipsilateral side, cephalad to the surgeon.

With the hip dislocated, the vastus lateralis is retracted with a Hohman retractor, and the lesser trochanter is exposed by subperiosteal dissection of tissues off the proximal femur [Figure 4b]. The lesser trochanter is a key landmark, since preoperative templating can identify the level of the calcar cut relative to this landmark, thereby allowing accurate limb length determination following the arthroplasty.^13^

The anticipated level of calcar cut is marked on the femur with a pen. The calcar cut is stopped about two-thirds of the way from medial to lateral, and the femoral head is reduced back in the socket. A second, lateral cut is placed vertically, just medial to the greater trochanter, and is completed with an osteotome. This technique will avoid the pitfall of inadvertent damage to the greater trochanter. With the femoral head free, the corkscrew is reinserted to extract the femoral head from the hip joint [Figure 4c].

#### Acetabular exposure

**Figure 5:**
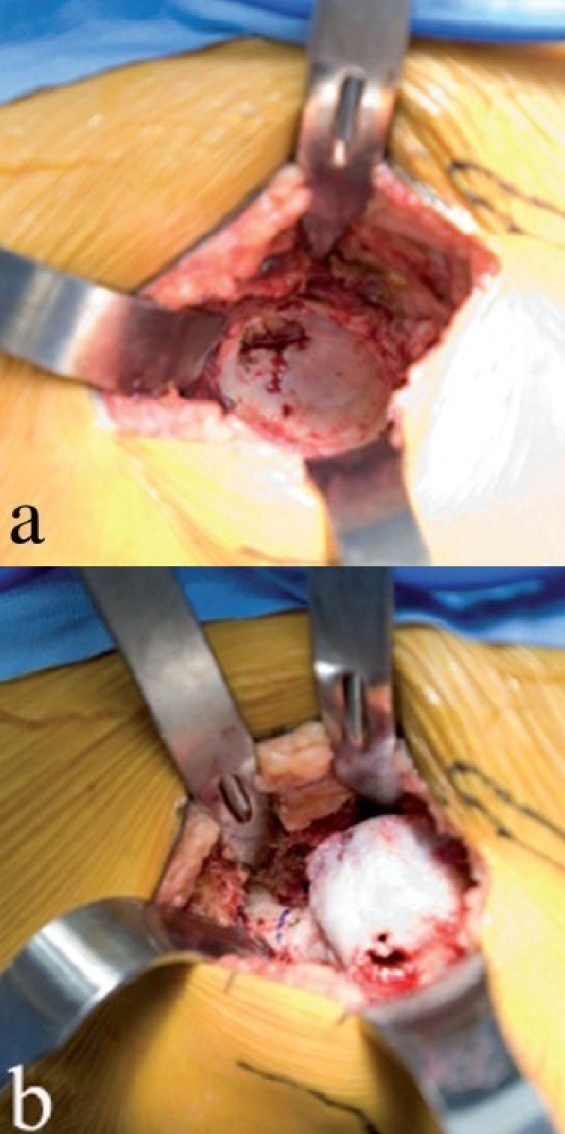
Excellent acetabular exposure (a) is possible with the anterior surgical approach to the left hip joint shown. Reaming (b) is done under direct vision, and X-ray control is not mandatory. Care should be taken to avoid levering the reamer handle on the tissues in the distal part of the incision

The lateral cobra retractor is repositioned inside the hip capsule to keep the tensor muscle retracted. A spiked Hohman retractor is placed on the anterior-inferior acetabular wall. A similar Hohman is placed on the anterior acetabulum, with the spike of the retractor resting directly on bone to avoid femoral nerve injury. With slight external rotation and gentle traction on the femur, acetabular exposure is typically excellent; circumferential visualization can help in removing osteophytes, reaming, and cup placement [Figure 5a].

**Figure 6:**
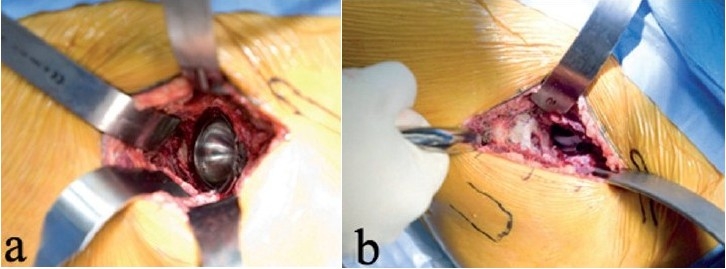
The acetabular shell placement is shown (a). Bearing insertion is easy, since adequate exposure is possible with the anterior approach. The surgeon's hand lifts up the femur as the leg is externally rotated to ensure that the proximal femur is not caught behind the acetabulum (b). One sharp Hohman is placed around the calcar, and the other is between the thick capsule at the top of the trochanter and the abductor muscles

Special side-cut acetabular reamers made for MIS-THA are used, although standard hemispherical reamers will suffice [Figure 5b]. A pitfall involves inadvertent levering of the reamer handle on the thigh. If this occurs, there is a risk of reaming out the anterior socket. The safe direction for reaming in the supine position is posterior, after the socket has been medialized. The acetabular notch should be identified since it can orient the surgeon to the medial wall. Cup placement is done with guides, and the bony anatomy can also confirm proper cup position [Figure 6a]. For surgeons accustomed to the lateral patient position, the supine position can mislead the surgeon into excessive cup anteversion, and vertical cup positioning. After acetabular screw fixation (if needed), osteophyte removal, and acetabular bearing insertion, attention is directed to femoral stem insertion.

#### Femoral exposure

The orthopedic table is equipped with a sterile bone hook designed to go around the proximal femur. This hook allows the surgeon to lift up the femur and estimate tissue tension, which varies by individual [Figure 6b]. The hook also allows the surgeon to ensure that the proximal femur is not caught behind the acetabulum. With the femur lifted up and laterally by the surgeon, the foot is externally rotated to about 90°, and dropped to the floor, thereby extending the femur. While keeping the proximal femur lifted, the motorized metal spar is manipulated with a footswitch and the hook is locked to the spar. From this point on, the surgeon should conceptualize the bone hook and external rotation of the leg as passive retraction devices, designed to facilitate exposure.

This is a key concept to understand because it will help avoid the pitfalls of inadvertent injury to the trochanter, ankle, or femur. Safe retraction during THA entails adequate mobilization of soft tissues first, followed by placement of a passive retractor. Forcing a retractor to compel surgical exposure is hazardous. This principle applies to the MIS-THA with the anterior approach also. The bone hook around the femur, and femur rotation and extension on the orthopedic table are passive retractors. In this sense, external rotation of the femur should be attempted only if the surgeon can rotate the ipsilateral knee manually. Similarly, manual elevation of the femur using the hook should precede lifting with the motorized elevator. When so used, the orthopedic table and bone hook will maintain the safe exposure that the surgeon gains by appropriate dissection; neither device is designed to compel exposure by force.

Preparation of the femoral canal should not commence until the proximal femur is adequately visualized. This requires a release of the thick hip capsule off the greater trochanter from anterior to posterior while protecting the abductors with a Hohman retractor. Additional femoral mobilization can be achieved by subperiosteal release of the short external rotators and the posterior hip capsule. This is possible by progressive external rotation of the femur. Prior exposure of the lesser trochanter will assist in this step. The exact amount of soft tissue release will vary by patient and anatomy, but by proceeding sequentially, satisfactory proximal femoral exposure can be gained in every patient. Short, obese, muscular patients are the most challenging, and in such cases, Trendelenburg positioning of the table can increase femoral extension, thereby lifting up the proximal femur for improved exposure.

In summary, the pitfall to avoid during femoral exposure is frustration and the urge to use the femoral hook, or employ vigorous external rotation to force adequate visualization. Proper exposure is achievable via a methodical, step-by-step process. The femoral hook and the orthopedic table should be thought of as passive retractors that can maintain the exposure safely achieved by judicious release of restraining structures.

#### Femoral preparation and stem insertion

**Figure 7:**
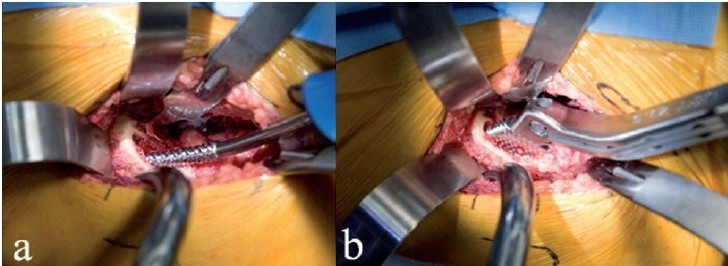
The curved awl is used to open the proximal femur under direct vision (a). A Hohman retractor protects the proximal incision. Rasping of the canal (b) is done under direct vision, with specially angled inserter handles

**Figure 8:**
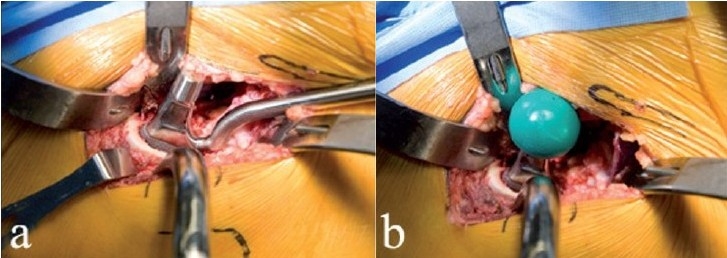
A ML taper stem (Zimmer) has been impacted in place (a) using an angled inserter handle. Since the calcar is visible, the danger of unrecognized proximal femur fracture is decreased. A trial head is assembled on the stem (b). Alternatively, the broach could have been left in place, thereby allowing trial reductions using various neck lengths and offset options

Once the proximal femur is adequately exposed, a Hohman retractor is positioned behind the greater trochanter, protecting the proximal part of the skin incision from femoral broaches. The canal is opened with a curved awl; the position of the knee can allow estimation of the femoral canal direction, thereby avoiding the pitfall of canal perforation [Figure 7a]. We have used uncemented press-fit femoral stems that require rasping of the femoral canal, without diaphyseal reaming. Specifically, the Corail stem (DePuy, Warsaw, IN), and the ML taper stem (Zimmer, Warsaw, IN) have been used with excellent results. Rasps, and stem inserters are mounted on instruments that are angled to clear the soft tissues proximally [Figures 7b, 8a].

Unrecognized calcar fracture is avoidable since the calcar is visualized during stem impaction. If an undisplaced calcar crack occurs, a cerclage wire can be placed around the proximal femur. Femoral head trials and neck trials can be inserted onto the femoral broaches to estimate femoral offset and leg lengths during trial reductions of the components [Figure 8b].

Leg lengths are measured by comparing the positions of the patellae on either leg, with the feet in neutral rotation. Preoperative templating and cutting the calcar at the estimated level can also ensure proper leg lengths during anterior MIS-THA. Manipulation of the leg during femoral preparation can be done by the scrub nurse; the training is relatively straightforward. Early in the surgeon's experience, a pin can be placed in the ipsilateral iliac crest; this will serve as a fixed landmark to measure any point on the anterior proximal femur, thereby allowing leg length measurement with certainty.

**Figure 9:**
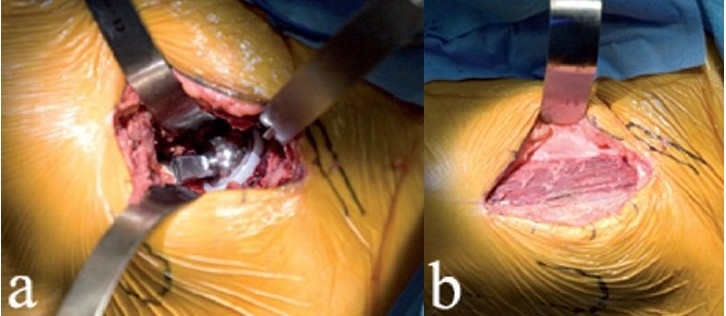
After final reduction, hip stability is assessed in maximal external rotation (a), and leg lengths can be confirmed. The approach is anatomically correct in that the overlying muscles fall in place and close the wound spontaneously as shown in this left hip (b). The fascia over the tensor is then reapproximated

Hip stability is assessed by maximally externally rotating the femur and checking for impingement or subluxation of the femoral head [Figure 9a]. This is relevant since anterior dissection logically destabilizes the hip anteriorly. Posterior stability can also be tested by removing the boot from the orthopedic table, but this has been unnecessary in our experience since it contributes no useful information. In combination with large diameter femoral heads, the procedure described here is inherently stable, and the risk of dislocation is almost zero.

#### Wound closure

The hip capsule flaps can be closed, and final wound closure can be over a drain, depending on surgeon preference. If a drain is used, the drain trocar should exit the lateral thigh since anteromedial drain placement increases the risk of neurovascular injury. The advantage of this surgical approach is evident during wound closure; the muscles of the anterior thigh simply fall into place as the retractors are removed [Figure 9b]. The fascia over the tensor muscle is repaired with sutures, followed by skin closure. Local anesthetic can be injected into the skin edges and subcutaneous tissues.

#### Rehabilitation

With spinal anesthesia, wound infiltration with local anesthetics, preemptive treatment of nausea and pain using preoperative medications, anterior MIS-THA is associated with little pain. Typically, patients do not require pain pumps or intramuscular narcotics; oral anti-inflammatory and narcotic medications are enough for comfort.

Physical therapy and patient mobilization are started on the day of surgery, or the next day. No hip precautions are needed, and patients can weight-bear with an assistive device for balance and safety. Patients can be sent home on the second postoperative day, with follow-up at four weeks postoperatively. During the month following surgery, home health visits ensure wound checks, compliance with warfarin therapy, suture removal, and hip exercises. No outpatient physical therapy is necessary. For prophylaxis against deep venous thrombosis, we have used dose-adjusted warfarin, compression stockings, early patient mobilization, and intermittent foot pumps that are applied intraoperatively.

## RESULTS

Using the technique described, 100 consecutive patients underwent anterior THA with uncemented components. We use this procedure routinely for all primary total hip replacements in our practice, and the results described here pertain to the first 100 patients who underwent this procedure. Mean patient age was 61 (range 33-91) years, and mean patient BMI was 29.8 (range 18.1-51.8). Outcomes were reviewed at a minimum follow-up period of 10 months.

**Figure 10:**
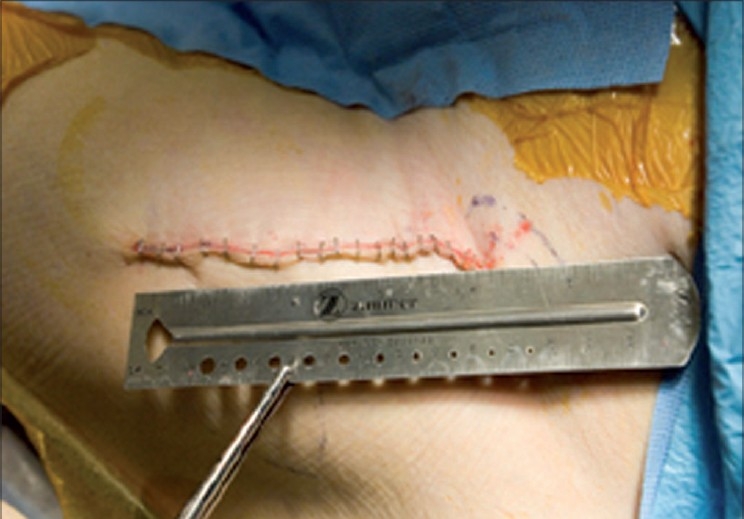
A typical incision on the left hip is shown following staple closure. An incision length of 8-15 cm is sufficient for all primary total hip replacements using the approach described

The mean duration of surgery was 53 min (range 34-87 min). The mean blood loss was 185 cc (range 65-630). The mean incision length [Figure 10] was 10.4 cm (range 7.8-13.7 cm). One patient suffered a non-fatal pulmonary embolism, and an undisplaced calcar fracture occurred in another patient. The calcar fracture was stabilized with a cerclage cable. This patient also developed increased wound drainage, possibly in response to the anticoagulants used to treat the pulmonary embolism; the wound drainage resolved spontaneously. No other skin maceration or other wound problems occurred.

No patient who was a candidate for a primary THA was excluded from the above series. Mean hospital stay was 2.4 days (range 1-5 days), and all patients were discharged home. All procedures were completed without intraoperative radiography.

Thigh numbness was present on objective testing in only four patients, and was clinically insignificant at the three-month visit. All patients had resumed their usual activities by four weeks after the procedure, and reported satisfaction with the outcome.

Component migration was defined as a >3 mm subsidence of the femoral implant, a >3° change in stem alignment relative to the femoral canal, or a >3° change in orientation of the metal cup. By these criteria, no components migrated in the present series. Mean acetabular component abduction was 42.3° (range 36.1-51.8°), mean cup anteversion on the true lateral radiograph was 15.2° (range 5.1-27.2°), and all but five femoral stems were within ±3° of varus/valgus alignment relative to the diaphyseal femoral shaft.

Three femoral stems were in >3° of varus relative to the diaphyseal femur on the anteroposterior (AP) hip radiograph. Varus stem positioning in these three was possibly the result of inadequate lateralization of the entry point during femoral preparation. The significance, if any, of this radiographic finding in the stem design used in this series is uncertain, especially since all stems were stable and none of the three patients reported hip pain.

## DISCUSSION

Complications such as intraoperative fractures, repeat surgery, nerve injury, excessive blood loss, prolonged operative times, skin maceration and component instability have been associated with MIS-THA, especially early in the learning curve.^13^–^16^ These adverse outcomes were not encountered in the first 100 patients who underwent anterior MIS-THA in the present series. In fairness, however, the present series reflects the experience of a high-volume arthroplasty surgeon who was thoroughly familiar with the Smith-Peterson surgical exposure. The surgeon had also undergone cadaver training and fellowship with an experienced mentor before attempting the first MIS-THA using an orthopedic table. Therefore, it is possible that the results reported here may not be reproducible in the hands of low-volume surgeons who lack experience with supine THA done through the anterior surgical approach.

### Advantages over MIS two-incision THA

In comparison to MIS two-incision THA, the method described here has practical advantages. The incision is placed more laterally on the thigh in anterior MIS-THA when compared to two-incision THA, thereby lessening the chance of injury to the lateral femoral cutaneous nerve. Two-incision MIS-THA can be associated with up to 25% incidence of injury to the lateral femoral cutaneous nerve palsy, even though most such injuries resolve with time.^13^ Since the incision is placed further laterally in anterior MIS-THA, the risk of femoral palsy may be reduced as well.

Blind femoral canal preparation and component placement during two-incision THA may lead to unrecognized muscle injury.^17^ A related complication is that of intramuscular hematoma from unrecognized bleeding in the gluteal muscles. During anterior MIS-THA, these risks are avoided since blind femoral canal preparation is not needed. Some cadaver evidence suggests that during anterior THA, intentional transection of piriformis and conjoint tendons is required to mobilize the femur, and that damage to the tensor and rectus femoris muscles can occur from retraction.^18^ In our experience, proper mobilization of the tensor and rectus femoris muscle, especially in the distal part of the surgical field will avoid retractor-induced damage. Furthermore, the mobilization of the piriformis and conjoint tendons is done subperiosteally, as the adherent capsule is peeled off the externally rotated femur. This type of release is less traumatic than cutting the short external rotators during posterior THA, and it does not result in posterior hip instability.

Intraoperative blood loss may be less with anterior MIS-THA when compared to the two-incision technique.^13^ One reason may be that during two-incision THA, the femur is placed over the other leg, and intramedullary blood runs down into the hip joint space as the femur is prepared. In contrast, during anterior MIS-THA the proximal femur is positioned above the knee joint. In addition to less blood loss, anterior MIS-THA is also associated with less risk of an unrecognized femur fracture; this is most likely related to the direct visualization of the femur, which is not always possible with two-incision MIS-THA.^16^

A practical advantage of anterior MIS-THA is that the broach is left in place and various neck/head combinations can be used to optimize the neck length and hip offset. With two-incision MIS-THA, the femoral stem has to be implanted first, and trial reductions with the broach alone are not possible.

Improved surgical exposure and the avoidance of blind femoral preparation make anterior MIS-THA more amenable to teaching in a training environment. Surgical exposure is relatively simple, and circumferential visualization of the bony socket is obtainable. Femoral instrumentation and preparation are done under direct visualization, and selected portions of the operation can be performed by supervised assistants while the surgeon maintains control of the procedure.

### Disadvantages of anterior MIS-THA

A disadvantage of the anterior approach is diminished access to the posterior column. If the patient has a deficient posterior acetabular wall from previous hardware or trauma, or if posterior acetabular augmentation is contemplated, the anterior exposure may be unsuitable. For MIS-THA with the anterior approach, the need for an orthopedic table may be another disadvantage. However, the investment may be worth the superior clinical outcomes, expeditious surgery, and easier surgical exposure during anterior MIS-THA. The table is also suitable for commonly performed orthopedic trauma procedures, such as the operative fixation of proximal femur fractures.

The lack of surgeon familiarity with the anterior approach may preclude widespread adoption of anterior MIS-THA, at least in the short term. New learning is associated with increased costs, risks, and new investments. Offsetting these considerations are the superior outcomes of anterior MIS-THA in terms of patient acceptance, reduced pain and disability, and rapid return to function. Proper learning of anterior MIS-THA should include a thorough familiarity with the anatomy of the anterior and lateral thigh, practice with cadaver dissection, and training with an experienced surgeon. The method illustrated in this report is used routinely in our practice for all uncomplicated primary total hip arthroplasty.

## CONCLUSIONS

Minimally invasive total hip arthroplasty based on the Smith-Petersen surgical interval provides the optimal combination of sufficient exposure, simplicity, safety, consistency, and preservation of muscle and tendons when compared with other methods of primary THA.^19^–^21^ Dissection is entirely within intermuscular planes, without disruption of tendinous insertions. Trial reduction and consistent component positioning are possible. Intraoperative fluoroscopy is an option for the surgeon. The supine patient position is more physiologic for the patient and anesthesiologist. With proper surgeon training, consistent and safe outcomes are possible.
